# *HOTAIR* Modulated Pathways in Early-Stage Breast Cancer Progression

**DOI:** 10.3389/fonc.2021.783211

**Published:** 2021-11-17

**Authors:** Martin C. Abba, María Laura Fabre, Jaeho Lee, Pradeep Tatineni, Hyunsuk Kil, C. Marcelo Aldaz

**Affiliations:** ^1^ Centro de Investigaciones Inmunológicas Básicas y Aplicadas (CINIBA), Facultad de Ciencias Médicas, Universidad Nacional de La Plata, La Plata, Argentina; ^2^ Department of Epigenetics and Molecular Carcinogenesis, The University of Texas M.D. Anderson Cancer Center, Houston, TX, United States

**Keywords:** *HOTAIR*, lncRNA, breast cancer, DCIS, proliferation, invasion

## Abstract

The long-non-coding HOX transcript antisense intergenic RNA (*HOTAIR*) was identified as significantly upregulated in breast ductal carcinoma *in situ* (DCIS). The aim of this study was to characterize the phenotypic effects and signaling pathways modulated by *HOTAIR* in early-stage breast cancer progression. We determined that *HOTAIR* induces premalignant phenotypic changes by increasing cell proliferation, migration, invasion and *in vivo* growth in normal and DCIS breast cell lines. Transcriptomic studies (RNA-seq) identified the main signaling pathways modulated by *HOTAIR* which include bioprocesses related to epithelial to mesenchymal transition, cell migration, extracellular matrix remodeling and activation of several signaling pathways (HIF1A, AP1 and FGFR). Similar pathways were identified as activated in primary invasive breast carcinomas with *HOTAIR* over-expression. We conclude that *HOTAIR* over-expression behaves as a positive regulator of cell growth and migration both in normal and DCIS breast cells involved with early-stage breast cancer progression.

## Introduction

Ductal carcinoma *in situ* (DCIS) is a premalignant lesion and non-obligate precursor to most invasive breast carcinomas (IBC). It has been estimated that more than one third of DCIS lesions have the potential to progress to invasive ductal carcinoma if left untreated (1). The reasons on why only some DCIS lesions progress to the invasive stage remain unclear. In a previous study, we performed the first comprehensive molecular profiling of pure high-grade (HG) DCIS lesions, thus identifying the main genomic, transcriptomic, methylation and gene pathway changes occurring at this pre-invasive breast cancer stage (2). RNA-seq profiling allowed us to identify HG-DCIS lesions with the most aggressive phenotypes, based on tumor intrinsic subtypes, proliferative, immune scores and in the activity of specific signaling pathways. Among the transcriptomic signatures of the most aggressive DCIS lesions, we identified the deregulated expression of almost 200 long-non-coding RNAs (lncRNAs), many of which might be associated with breast cancer progression. HOX transcript antisense intergenic RNA (*HOTAIR*) was one of the most significantly upregulated lncRNAs in aggressive DCIS lesions (2). LncRNAs are defined as non-coding RNAs exceeding 200 nucleotides in length and without evident protein coding functions (3). Over ten thousand lncRNAs have been annotated in the human genome, and although they have been increasingly implicated in neoplastic diseases, only a few have been functionally characterized (4).

*HOTAIR* belongs to the first lncRNAs described as aberrantly expressed in invasive breast carcinomas. Since its identification in breast cancer, *HOTAIR* overexpression has been reported in almost all solid tumor sites (5). *HOTAIR* is transcribed from the anti-sense strand of the *HOXC* gene cluster located on chromosome 12q13.13 and it serves as scaffold to epigenetically repress expression of the more distal *HOXD* gene cluster and genes in other chromosomes (6). *HOTAIR* is able to bind two different chromatin modifiers: the Polycomb Repressive Complex (PRC2) at the 5’ end, and the Lysine-Specific histone Demethylase 1 complex (LSD1) at the 3’ end (6, 7). Hence, *HOTAIR* has bifunctional modulation on chromatin status epigenetically repressing the transcription of their target genes. In addition, *HOTAIR* is also implicated in post-transcriptional and post-translational modulation by interaction with multiple miRNAs (e.g. *miR-7*, *miR-148a*, *miR-204*) or binding to E3 ubiquitin ligases, such as Mex3b and Dzip3, and promoting target degradation (8, 9). *HOTAIR* overexpression has been extensively described in primary and metastatic breast cancer. Early studies associated overexpression of this lncRNA in primary breast carcinomas with high metastatic potential and poor overall patient survival (10). Further studies postulated *HOTAIR* upregulation as a prognostic marker of lymph node metastases in ER-negative breast cancer patients (11). *HOTAIR* has been shown to modulate critical molecular pathways related to breast cancer development and progression such as autophagy, epithelial mesenchymal transition (EMT), and drug resistance (12). *HOTAIR* overexpression increases the invasive ability of breast cancer cells *in vitro* and *in vivo* (10). Notably, in murine xenograft models, *HOTAIR* knockout can reduce tumor growth *in vivo*. Thus, *HOTAIR* has been postulated as putative breast cancer oncogene (5).

Here we characterized for the first time the phenotypic and molecular effects of *HOTAIR* overexpression in non-invasive breast cancer models. Overall, we also demonstrated the relevance of its pro-oncogenic behavior at early-stages of breast cancer progression.

## Material And Methods

### Cell Lines, Cell Culture

MCF10A cell line was obtained from the American Type Culture Collection (#CRL-10318; ATCC, VA, USA) and validated by DNA fingerprinting. MCF10A cells were cultured in Dulbecco’s Modified Eagle Medium F-12 (DMEM/F-12, Sigma-Aldrich) supplemented with 5% horse serum, 20 ng/mL epidermal growth factor (Sigma-Aldrich), 100 µg/mL hydrocortisone (Sigma-Aldrich), 10 µg/mL insulin (Sigma-Aldrich), 100 ng/mL cholera toxin (Sigma-Aldrich) and 100 U/ml penicillin - 100 μg/ml streptomycin (Sigma-Aldrich). MCF10 DCIS.COM (hereafter DCIS.COM) cells were a kind gift from Dr. Daniel Medina (13) and were maintained in DMEM/F-12 supplemented with 5% horse serum. Cell lines were maintained to 37°C with 5% CO2.

### Stable *HOTAIR* Expressing Cells

The full-length sequence of *HOTAIR* (2146 bp spanning six exons) was obtained from Addgene (Plasmid #26110, Watertown, MA USA), sequenced verified and subsequently cloned into the pCDH lentiviral expression vector. Virus particles were produced using packaging line Lenti-X 293T (Takara Bio, CA USA). Normal breast epithelial cell lines MCF10A and DCIS cell line DCIS.COM were stably transduced and selected with 10µg/ml puromycin.

### Cell Proliferation, Clonal Growth and Migration Assays

MCF10A stably transduced to overexpress *HOTAIR* or an empty vector control were plated (1,000 cells per well) on 96 well plates in triplicate and cell proliferation was determined by means of the colorimetric MTT assay kit (Cell Proliferation Kit, Roche) and measuring optical density (OD). For clonal growth assays, MCF10A stably transduced to overexpress *HOTAIR* or vector control were plated at clonal density (500 cells/dish) in individual wells of 6-well plates and maintained in adequate media as described above. After 9 days of growth, cells were fixed and colonies stained with crystal violet. Digital images of individual wells were obtained and used to determine the number and area of growing colonies using ImageJ software. Transwell migration assays were performed using standard Boyden chambers containing 12 μm pore divider membranes, 5% FBS was used in the lower chamber as chemoattractant. Statistical significance was determined using Mann-Whitney-Wilcoxon test.

### Mammary Intraductal DCIS Xenograft Model (MIND)

DCIS.COM stably transduced cells with *HOTAIR* (n=3) or empty vector as control (n=3 mice) were inoculated *via* the nipple using a 30-gauge Hamilton syringe into the intact main mammary duct of both inguinal mammary glands of female SCID mice 6-8 wks of age. Tumor growth was monitored, and after an observation period of 10 wks. post injection, mice were euthanized and both inguinal mammary glands were dissected. Xenografts of wild-type DCIS.COM cell line result in the formation of DCIS-like tumors but do not invade (13).

### RNA-Seq Data Analysis

MCF10A and DCIS.COM stably transduced cells were used for RNA isolation from subconfluent plates using the RNeasy kit (Qiagen, CA, USA). RNA concentration and integrity were measured on an Agilent 2100 Bioanalyzer (Agilent Technologies). Only RNA samples with RNA integrity values (RIN) over 8.0 were considered for subsequent analysis. RNA-seq library construction was performed using the ScriptSeq v2 RNA-seq Library Preparation Kit (Epicentre) according to the manufacturer’s protocol. We performed 76 nt paired-end sequencing using an Illumina HiSeq2000 platform and ~20 million reads per sample were obtained. The short-sequenced reads were mapped to the human reference genome (hg19) by the splice junction aligner Rsubread package. We employed several R/Bioconductor packages to accurately calculate the gene expression abundance at the whole-genome level using the aligned records (BAM files) and to identify differentially expressed genes between cells stably transduced with *HOTAIR* and empty vector. Briefly, the number of reads mapped to each gene based on the UCSC.hg19.KnownGene database were counted, reported and annotated using the featureCounts and org.Hs.eg.db packages. Data are available at GEO under accession number GSE183058. To identify differentially expressed genes (log2 fold change [FC] > ± 1.5, False Discovery Rate [FDR] <0.05) between the empty vector and *HOTAIR* overexpressing counterparts, we utilized the edgeR Bioconductor package based on the normalized log2 based count per million values. For functional enrichment analyses, we used R/Bioconductor clusterProfiler package and the InnateDB resource (http://www.innatedb.com/) based on the list of dysregulated transcripts. Data integration and visualization of differentially expressed transcripts were done with R/Bioconductor and the MultiExperiment Viewer software (MeV v4.9).

### *In Silico* Analysis of *HOTAIR* in Normal and Breast Cancer

Pre-processed *HOTAIR* expression profiles among five early-stage breast cancer datasets: GSE69994 (2), GSE59246 (14), GSE41228 (15), GSE66301 (16) and GSE47462 (17) were obtained from GEO and analyzed using R software. In addition, pre-processed *HOTAIR* RNA-seq expression levels among primary breast carcinomas with intrinsic subtype data and their integrated pathway activities (pathway activity – z score of 1387 constituent PARADIGM pathways) were obtained from the TCGA Breast Cancer (BRCA) dataset through the UCSC Xena browser (http://xena.ucsc.edu/). The PARADIGM algorithm integrates pathway, expression, and copy number data to infer activation of pathway features within a superimposed pathway network structure extracted from NCI-PID, BioCarta, and Reactome (18). Briefly, primary breast carcinomas (n = 1097) were divided into low (n=191) or high (n=392) *HOTAIR* expression levels according to the StepMiner one-step algorithm (19). These two groups were then compared at their integrated pathway activities to identify the most relevant signaling pathways associated with *HOTAIR* expression using the T-test (p-adj. < 0.01) with MultiExperiment Viewer Software (MeV 4.9). Statistical analysis was performed using the computing environment R.

## Results And Discussion

### *HOTAIR* Overexpression in Early-Stage Breast Cancer

**Figure 1:**
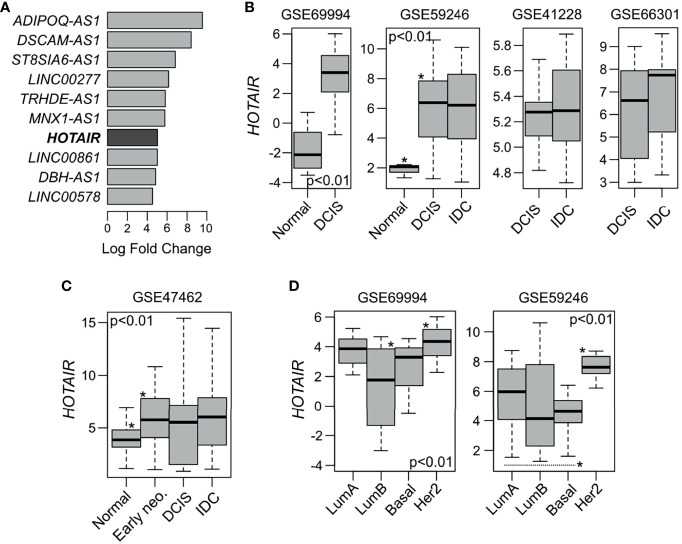
*HOTAIR* expression in normal, pre-invasive and invasive breast samples. **(A)** Top ten most upmodulated lncRNAs in DCIS lesions compared with normal breast samples according to GSE69994 study (2). **(B)** *In silico HOTAIR* expression analysis among normal, DCIS and IBC samples obtained from four independent GEO dataset (2, 14–16). *HOTAIR* expression was significant upregulated in DCIS and IBC samples compared with normal samples (p < 0.01), while non-significant differences were observed between DCIS and IBC cases (p > 0.05). **(C)** *HOTAIR* expression analysis among normal and DCIS precursor lesions (early neo.) such as columnar cell lesions and atypical ductal hyperplasia, obtained from GSE47462 dataset (17). **(D)** *HOTAIR* expression analysis across DCIS intrinsic subtype obtained from two independent GEO datasets (2, 14). ANOVA or T-test were used to compare the *HOTAIR* expression among groups. *Statistical significance differences.

In a previous study, we performed a comprehensive molecular profiling of ‘pure’ high-grade DCIS lesions, providing the first catalogue of genomic, transcriptomic, methylation and gene pathway changes occurring at this pre-invasive breast cancer stage (2). Among the most significantly upregulated lncRNAs we found *HOTAIR* (fold change (FC) = 32.7; false discovery rate (FDR) < 0.0001) when DCIS were compared with normal breast tissue (**Figure 1A**). Therefore, we hypothesize that *HOTAIR* might have a relevant role also in early-stage breast development and not just in later stages of tumor progression as previously described. In this study we characterized the molecular and phenotypic effects of *HOTAIR* expression in normal and non-invasive breast cancer models. *In silico* analysis of *HOTAIR* expression among five early-stage breast cancer datasets obtained from Gene Expression Omnibus (GEO) showed significant upregulation of this transcript in DCIS and IBC when compared to normal samples (p < 0.01; **Figure 1B**). However, non-significant differences were observed in *HOTAIR* expression levels when DCIS was compared with IBC samples as seen in analyses of three independent DCIS-IBC datasets (p > 0.05; **Figure 1B**) (14–16). *HOTAIR* expression levels were also evaluated in normal and early stage neoplasias (including columnar cell lesions and atypical ductal hyperplasia) obtained from the GSE47462 dataset (17). Interestingly, the DCIS precursor lesions (described as early-neoplasia in **Figure 1C**), showed significant *HOTAIR* overexpression when compared with normal samples (p<0.001; **Figure 1C**). *HOTAIR* expression was also compared across DCIS intrinsic subtypes in two independent datasets (2, 14). HER2 and luminal A DCIS intrinsic subtypes showed significantly higher *HOTAIR* expression levels compared with the luminal B and basal-like subtypes (p<0.01; **Figure 1D**).

*HOTAIR* expression is modulated by multiple signaling pathways. Its promoter sequence contains binding sites for diverse transcription factors, such as estrogen response elements (EREs), hypoxia response elements (HREs), AP1 response elements (TREs) among others (20). *HOTAIR* expression can be induced by estradiol (E2) in an estrogen receptor dependent manner through EREs or independent *via* interaction with G-protein-coupled estrogen receptor-1 (GPER) (21, 22). HER2 has also been recently described as an activator of *HOTAIR* expression by acting on the effector mitogen-activated protein kinase (MAPK) in primary invasive breast carcinomas and invasive breast cancer cells (23). In agreement with these observations, the HER2 DCIS intrinsic subtype appears as the group with highest *HOTAIR* expression levels followed by the E2/ER responsive luminal A subtype (**Figure 1D**).

Overall, these data suggest that *HOTAIR* over-expression might be a critical molecular event promoting breast cancer development at early pre-invasive stages, remaining up-modulated in invasive and metastatic carcinomas in specific molecular subtypes.

### Transcriptome Analysis of *HOTAIR* Overexpressing Cells

**Figure 2:**
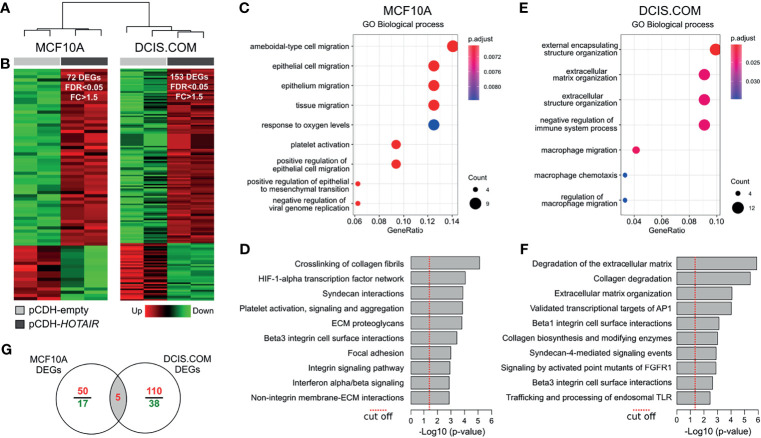
Transcriptomic analysis of *HOTAIR* overexpressing cells. **(A)** Hierarchical clustering of MCF10A and DCIS.COM stably transduced cells with either vector control (pCDH-empty) or lentivirus expressing *HOTAIR* (pCDH-*HOTAIR*) based on RNA-seq profiles. **(B)** Heat map representation of the differentially expressed genes (DEG) obtained by RNA-seq analysis (FDR<0.05; FC>1.5). Red represents upregulated genes and green downregulated genes. **(C, D)** Functional enrichment analysis of GO biological processes and pathways identified as affected by expression of *HOTAIR* in MCF10A cells. **(E, F)** Functional enrichment analysis of GO biological processes and pathways identified as affected by expression of *HOTAIR* in DCIS.COM cells. **(G)** Venn diagram of transcripts commonly modulated among MCF10 and DCIS.COM cells stably transduced with *HOTAIR*.

To better understand the mechanism of action of *HOTAIR* and their phenotypic impact in normal and DCIS cells, MCF10A and DCIS.COM cells were stably transduced for *HOTAIR* overexpression for further transcriptomic, *in vitro* and *in vivo* characterization. Whole-transcriptome unsupervised analysis from RNA-Seq data demonstrates a clear segregation of transduced cells in MCF10A and DCIS.COM groups (**Figure 2A**).

RNA-seq analysis of MCF10A cells identified 72 differentially expressed genes (DEG) of which 55 were upregulated and 17 were downregulated comparing *HOTAIR* expressing cells with the empty-vector cells (FC ≥ 1.5; FDR ≤ 0.05; **Figure 2B** and **Supplementary Data 1**). Functional enrichment analysis of DEG in MCF10A cells indicated a robust association with positive regulation of cell migration and EMT gene ontology (GO) biological processes (p < 0.001; **Figure 2C**). Remarkably, extracellular matrix (ECM) interaction/remodeling and HIF1A transcription factor signaling were among the most significantly modulated pathways in MCF10A cells stably transduced with *HOTAIR* (**Figure 2D**). Among the upregulated genes in MCF10A, we found *AXL* (*AXL receptor tyrosine kinase*), *ANGPTL4* (*Angiopoietin like 4*), *MALAT1* (*Metastasis associated lung adenocarcinoma transcript 1*), *VIM* (*Vimentin*) and *CDH2* (*Cadherin 2*). These genes are involved in pro-tumorigenic processes including EMT, cell migration, invasion and stemness (24–26). EMT is a dynamic and reversible process modulated by epigenetic regulators (PRC2, NuRD, LSD1 and PHF2) and gene expression changes (27, 28). *HOTAIR* interacts with PRC2 to trigger H3K27 methylation of EMT gene promoters (10, 29).

Furthermore, a recent study has shown that *HOTAIR* negatively regulates the function of LSD1 in maintaining epithelial identity demonstrating that most of the transcriptome changes induced by *HOTAIR* require both PRC2- and LSD1-interacting domains (30). In this sense, the upmodulation of mesenchymal markers such as *VIM* and *CDH2* in MCF10A *HOTAIR* transduced cells, clearly suggests *HOTAIR* involvement in EMT regulation at early stages of breast cancer progression. As mentioned, the lncRNA *MALAT1* was also detected as upregulated in association with *HOTAIR* overexpresion in MCF10A cells. This lncRNA was initially identified as upregulated in primary non-small cell lung cancer cells with higher metastasis ability and subsequently associated with other tumor types (24, 31). Recently, *MALAT1* was identified as a hypoxia-induced transcript that could promote cellular migration and proliferation of breast cancer cells (32). Interestingly, the HIF-1 alpha transcription factor network was among the most significantly enriched pathways in MCF10A *HOTAIR* transduced cells suggesting a cooperative role between both oncogenic lncRNAs.

In DCIS.COM cells *HOTAIR* overexpression caused the deregulation of 153 genes, of which 115 were upregulated and 38 were downregulated (FC > 1.5; FDR < 0.05; **Figure 2B** and **Supplementary Data 1**). Functional enrichment analysis of DEG in DCIS.COM showed a significant enrichment of ECM organization and immune related GO biofunctions (**Figure 2E**). Consistently, ECM/Collagen degradation and AP1 and FGFR1 signaling pathways were significantly dysregulated in DCIS.COM cells stably transduced with *HOTAIR* (**Figure 2F**). Among the upregulated genes in DCIS.COM, we found several matrix metallopeptidases (e.g.: *MMP2*, *MMP14*, *MMP28*), and fibrogenic ECM (e.g.: *COL7A1*, *COL9A3*, *COL16A1*, *COL17A1*) and Beta1/3 integrin related genes (e.g.: *COL7A1*, *MDK*, *PLAU*). The ECM is composed of a complex meshwork of highly cross-linked components, including fibrous proteins, glycoproteins, proteoglycans, and polysaccharides. Matrix metalloproteinases are zinc-dependent endopeptidases involved in ECM degradation and tissue remodeling. These endopeptidases are capable of degrading both the ECM and basement membrane, physical barriers that prevent expanding growth and migration of cancer cells (33). In addition, several studies have involved the high collagen and integrins expression levels with the tumor stroma-associated fibrosis (also called desmoplasia), a process that promotes tumor cells migration and metastasis (34). In this sense, increased *HOTAIR* expression in DCIS could facilitate acquiring the invasiveness capability to progress to the malignant stages. Despite the small number of genes commonly modulated between MCF10A and DCIS.COM cells (*HOTAIR*, *GNG2*, *ENPP2*, *PPFIA4* and *NDRG1*) (**Figure 2G**), several pathways related with ECM organization, collagen degradation, and Beta integrin cell surface interactions were commonly modulated between normal and DCIS *HOTAIR* transduced cells (**Supplementary Data 1**).

### *HOTAIR* Overexpression Promotes Proliferation, Migration, and Invasion of Normal Breast Epithelial Cells

**Figure 3:**
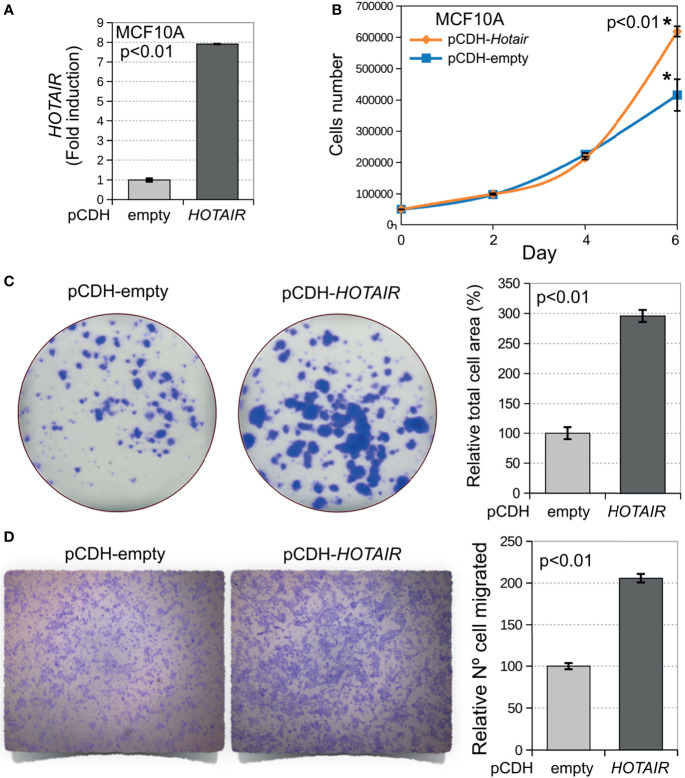
Stable overexpression of *HOTAIR* induces increased cell proliferation, colony growth and invasion in normal breast cells. **(A)** Levels of *HOTAIR* expression in stably transduced MCF10A cells based on their RNA-seq profiles (log2 CPM). **(B)** Stable overexpression of *HOTAIR* increases cell proliferation in normal breast cells (p < 0.01). Cells were plated 1,000 cells per well on 96 well plates in triplicate and cell proliferation was determined by means of the MTT colorimetric assay and measuring optical density (OD). **(C)** Cells stably transduced with lentivirus expressing *HOTAIR* or vector control were plated at clonal density in 6-well plates. Cells were allowed to grow for 9 days, fixed and stained with crystal violet. Bar chart displays increased area occupied by colonies for *HOTAIR* stably transduced cells compared with vector control. **(D)** Transwell migration assay of DCIS.COM cells stably transduced with *HOTAIR*. On the left comparative pictures of cells that migrated through the membrane, on the right bar chart of the relative numbers of cells per membrane for *HOTAIR* stably transduced cells compared with vector control (p < 0.01). Statistical significance was determined using Mann-Whitney-Wilcoxon test. *Statistical significance differences.

To investigate the phenotypic impact of *HOTAIR* overexpression in normal breast epithelial cells, we conducted cell proliferation, colony formation, and transwell migration assays on stably transduced MCF10A cells (**Figure 3A**). We first determined the effects of stable *HOTAIR* expression on cell proliferation by means of the MTT assay. As can be observed in **Figure 3B**, stable *HOTAIR* expression behaved as a pro-oncogenic stimulus inducing increased cell proliferation in normal breast cells after a week of cell culture (p < 0.01). The positive effect of *HOTAIR* on cell proliferation was further confirmed by means of colony formation assays. MCF10A cells stably transduced to overexpress *HOTAIR* displayed dramatic increase in colony growth when seeded at clonal density (**Figure 3C**). MCF10A cell line showed increased percentage area covered by colonies (p < 0.01) indicating increased cell growth (cell proliferation) as consequence of *HOTAIR* overexpression. Furthermore, MCF10A cells stably transduced with *HOTAIR* encoding lentivirus were also characterized by effects in the transwell migration assay (p < 0.01) (**Figure 3D**). The described results demonstrate that *HOTAIR* indeed behaves as a positive regulator of cell growth and migration in normal breast cells.

### *HOTAIR* Overexpression Promotes *In Vivo* Invasive Tumor Growth of DCIS Cells

**Figure 4:**
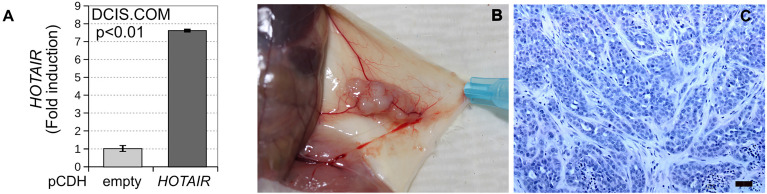
DCIS.COM stably transduced cells with either vector control or lentivirus expressing *HOTAIR* were compared using the *in vivo* MIND model. **(A)** *HOTAIR* expression levels in stably transduced DCIS.COM cells based on their RNA-seq profiles (log2 CPM). **(B)** DCIS growth was detected among injected mice. **(C)** Representative invasive ductal carcinoma (10X H&E staining) induced by DCIS.COM cells stably transduced with *HOTAIR*. Scale bar: 200 μm.

To investigate the effects of *HOTAIR* on the *in vivo* progression of the stably transduced DCIS.COM cell line (**Figure 4A**), the mammary intraductal DCIS model (MIND) was employed (13). Briefly, the MIND assay consists in the inoculation with DCIS.COM cells (*HOTAIR* transduced or empty vector) *via* the nipple into the intact main mammary duct of both inguinal mammary glands of female NSG mice. Behbod et al. have described that DCIS.COM cells when injected *via* intra-nipple in NSG mice, grow intraductally and do not invade, thus mimicking the non-invasive features of DCIS growth and lesions look histologically almost identical to clinical DCIS (13). DCIS.COM cells stably transduced with *HOTAIR* produced *in vivo* growth and development of invasive lesions in 2 out of 3 injected mice (**Figures 4B, C**), while DCIS.COM invasive growth was not observed in mice injected with DCIS.COM cells transduced with empty vector (n= 3). These data suggest that *HOTAIR* overexpression may behave as a driver of growth *in vitro* and *in vivo* at premalignant stages of breast cancer progression.

### Conserved *HOTAIR* Modulated Pathways Among Pre-Invasive and Invasive Stages

**Figure 5:**
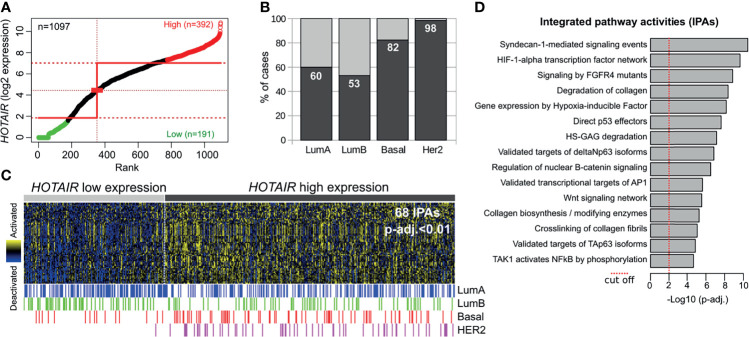
*HOTAIR* expression and pathway activity analysis in primary invasive breast carcinomas. **(A)** Primary breast carcinomas were divided into *HOTAIR* low or high expression levels based on the StepMiner algorithm using TCGA RNA-seq datasets obtained from the UCSC Xena resource (https://xenabrowser.net/). **(B)** Percentage of cases with high or low *HOTAIR* expression among intrinsic subtypes showing a consistent up-regulation in basal-like and HER2 subtypes compared with luminal-like tumors. **(C)** Heat map with the 68 significantly activated PARADIGM Integrated Pathways Activities (IPAs) among *HOTAIR* high expression tumors (p-adj.<0.01). **(D)** Bar plot of the top fifteen activated pathways determined by PARADIGM algorithm in primary invasive breast carcinomas with *HOTAIR* overexpression.

To further evaluate the relevance of the *HOTAIR* expression and their modulated pathways identified in normal and pre-invasive models with the invasive stage, we performed an *in silico* analysis on invasive breast carcinomas obtained from TCGA (n=1097). The Step-miner algorithm (19) allowed us to identify primary tumors with high (n=392) or low (n=191) *HOTAIR* expression (**Figure 5A**). Interestingly, a significantly larger number of tumors with high *HOTAIR* expression were detected in HER2+ (98%) and basal-like (82%) subtypes compared with luminal A (60%) and luminal B (53%) breast cancer subtypes (p < 0.0001; **Figures 5B, C**). These results are in agreement with higher *HOTAIR* expression levels detected in HER2+ DCIS than any of the other subtypes (**Figure 1B**). Analysis of pathway-based representation analysis (PARADIGM) identified 68 activated signaling pathways in invasive carcinomas with high *HOTAIR* expression compared with low expression counterparts (p-adj. < 0.01; **Figure 5C**). Interestingly, several of the activated signaling pathways identified in invasive carcinomas with high *HOTAIR* expression (**Figure 5D**) were detected in normal and DCIS *HOTAIR* stably transduced cells such as: Syndecan signaling, HIF1A transcription factor network, FGFR signaling, degradation of collagen, AP1 transcriptional targets, among others (**Supplementary Data 2**). However, other activities such as p53/p63, Wnt and nuclear B-catenin signaling were only detected in the invasive stage associated with *HOTAIR* overexpression. Nevertheless, our results revealed that multiple signaling pathways associated with *HOTAIR* overexpression in invasive breast carcinomas were also modulated in normal and DCIS *HOTAIR* transduced cells. Overall, the comparative transcriptomic analysis suggests that *HOTAIR* is probably a critical mediator of the EMT, cell migration, and ECM remodeling programs to drive breast cancer progression at premalignant stages.

In conclusion, the described results indicate that *HOTAIR* overexpression induces premalignant phenotypic changes in normal breast epithelial and DCIS cells compatible with the necessary steps towards malignancy, such as increase in cell proliferation, migration and invasion. In agreement with the *in vitro* and *in vivo* observations, we identified that *HOTAIR* upmodulates the expression of transcripts associated with the epithelial to mesenchymal transition, cell migration, and extracellular matrix degradation among other bioprocesses. Finally, *HOTAIR* overexpression was significantly associated with HER2+ DCIS and IBC subtypes. Further mechanistic characterization of *HOTAIR* in preinvasive *in vitro* and *in vivo* models may provide insights into how this oncogenic lncRNA could contribute to the early stages of breast cancer development and progression.

## Data Availability Statement

The transcriptomic data are available at GEO under accession number GSE183058. https://www.ncbi.nlm.nih.gov/geo/query/acc.cgi?acc=GSE183058.

## Ethics Statement

The study was conducted according to the guidelines of the Declaration of Helsinki, and approved by the Institutional Review Board of UT-MDACC.

## Author Contributions

MCA and CMA contributed the conception of the project and the design of all experiments. Experiments were conducted by JL, PT, and HK. MCA and MF carried out all bioinformatic analyses. MCA and CMA wrote the main body of the manuscript. All authors contributed to the article and approved the submitted version.

## Funding

This work was supported by the Office of the Assistant Secretary of Defense for Health Affairs through the Breast Cancer Research Program under Award No. W81XWH-16-1-0027, BC150021, the MD Anderson Cancer Center Support Grant P30 NIH CA16672, and the CPRIT Core Facility Support Grant RP170002 to CMA, and the Argentine National Agency of Scientific and Technological Promotion, grant PICT-2018-01403 to MCA.

## Conflict of Interest

The authors declare that the research was conducted in the absence of any commercial or financial relationships that could be construed as a potential conflict of interest.

## Publisher’s Note

All claims expressed in this article are solely those of the authors and do not necessarily represent those of their affiliated organizations, or those of the publisher, the editors and the reviewers. Any product that may be evaluated in this article, or claim that may be made by its manufacturer, is not guaranteed or endorsed by the publisher.

## Supplementary Material

Supplementary Data Sheet 1 Differentially expressed genes and deregulated pathways in MCF10A and DCIS.COM stably transduced cells.

Supplementary Data Sheet 2 Activated pathways in *HOTAIR* high vs. low expression invasive breast carcinomas.
